# Dual-Atom Catalysts for the Oxygen Reduction Reaction: Unraveling Atomic Structures under Reaction Conditions

**DOI:** 10.1021/jacs.5c04776

**Published:** 2025-05-23

**Authors:** Courtney Brea, Guoxiang Hu

**Affiliations:** † Department of Chemistry and Biochemistry, Queens College, 14781 City University of New York, New York, New York 11367, United States; ‡ The Graduate Center, City University of New York, New York, New York 10016, United States; § School of Materials Science and Engineering, 1372 Georgia Institute of Technology, Atlanta, Georgia 30332, United States; ∥ School of Chemistry and Biochemistry, Georgia Institute of Technology, Atlanta, Georgia 30332, United States

## Abstract

Metal–nitrogen–carbon (M–N–C, *M* = Mn, Fe, Co, Ni, Cu, Zn, and Pt) dual-atom catalysts (DACs) show great potential for the oxygen reduction reaction (ORR) at the cathode of proton exchange membrane fuel cells (PEMFCs). During catalytic reactions, multiple reactants and intermediates interact with the active sites, yet understanding their dynamic structural evolution under the operating conditions remains challenging. In this study, we analyze 186 heteronuclear FeM–N–C DACs using *ab initio* thermodynamic phase diagrams and find that OH-ligated structures become predominant at higher applied potentials. This indicates that catalytic activity is governed by electrochemically modified metal sites rather than by the bare structures. We further investigate the catalytic mechanism of these ligated structures and reveal that the ORR limiting potential can be efficiently predicted from the phase diagrams. Among the 186 DACs studied, 29 were found to outperform Pt-based catalysts, with FeCo–N–C DACs demonstrating the highest activity. Our computational predictions align well with experimental observations, highlighting the crucial role of dynamic structural changes under reaction conditions in enhancing the electrocatalytic performance of DACs.

## Graphical abstract

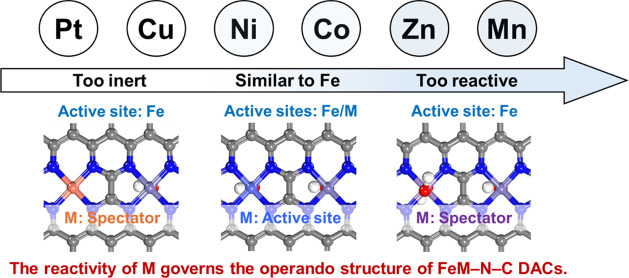

## Introduction

Electrocatalysis plays a pivotal role in many sustainable energy technologies, where efficient catalysts are essential. One prominent example is the proton exchange membrane fuel cell (PEMFC),^1–3^ which uses hydrogen and oxygen (from air) to generate electricity, producing only water and heat as byproducts. In this system, the oxygen reduction reaction (ORR) at the cathode plays an important role, as it faces a significantly higher kinetic barrier compared to the hydrogen oxidation reaction (HOR) at the anode, thereby limiting the overall efficiency of the fuel cell.^4,5^ Currently, the most effective catalysts for ORR are platinum (Pt) or Pt alloys; however, their high cost and low abundance pose significant obstacles to large-scale implementation.^6–9^

Iron–nitrogen–carbon (Fe–N–C) single-atom catalysts (SACs) offer a promising alternative to Pt for the ORR due to their low cost, high abundance, and comparable catalytic activity to Pt.^10–18^ Despite significant advancements, Fe–N–C SACs still face challenges related to stability and activity,^19,20^ primarily due to their susceptibility to demetalation^21–23^ and the lack of synergistic sites for ORR, which involves multiple reaction intermediates. Recent experimental studies suggest that incorporating a second transition metal to create dual-atom catalysts (DACs) can further enhance ORR performance on Fe–N–C SACs.^24–31^ In these catalysts, the neighboring metal sites can work synergistically to enhance the activity. In addition, their interactions help stabilize individual metal sites, preventing the demetalation.^24,27,32^ However, it remains a challenge to understand the structure–property relationship at the atomic level, which is crucial for the rational design of these catalysts.

While experimental studies have provided evidence for the existence of atomically dispersed dual-atom pairs, including insights into their coordination environment and spatial distribution, fully understanding their atomic structure under reaction conditions still presents challenges.^33,34^ One key question is whether the active sites of DACs remain in their bare, pristine form or become ligated by the reactants or intermediates during operation. The adsorption of various species, such as oxygen-containing intermediates, can significantly alter the electronic structure of the catalytic centers, thereby influencing their reactivity and stability. For example, *OH ligands derived from water dissociation have been demonstrated to play a critical role in modulating the electronic structure of the active sites of SACs and DACs.^35–39^ However, due to the limited availability of in situ and operando experimental techniques that can probe atomic-level transformations in real time, the true active sites in DACs remain largely unknown.

Previously, we investigated the structural evolution of FeCu–N–C DACs under an applied electrode potential, and our findings suggest that *OH species preferentially occupy the Fe active site at elevated potentials.^40^ In this work, to gain deeper insights into the potential-dependent atomic structures of all experimentally synthesized DACs, we examine a broader range of surface coverages from 1/2 to 2 ML *OH across 186 DAC structures using *ab initio* phase diagrams. A key focus is determining whether both metal sites in these catalysts undergo induced modifications in response to the applied potential. We further investigate the ORR mechanisms using the potential-modulated structures to predict their catalytic activity. Our study offers an atomistic-level analysis of the structural evolution of DACs under operating conditions, highlighting the critical importance of identifying their true active sites during reaction to predict and enhance their performance for ORR.

## Results and Discussion

Building on our previous work,^40^ we generated all possible configurations for the dual metal sites in DACs. As shown in Figure 1a, this was done by tiling the hexagonal lattice of graphene with the first metal at the origin point (0, 0). Each configuration can then be labeled by the coordinate of the second metal site. Figure 1a shows three representative configurations (0, 1), (1, 2), and (3, 3) from this method, while the atomic structures for all 31 configurations are provided in Figure S1. With this method, we successfully captured all previously reported structural configurations,^41,42^ in addition to the new ones. We applied this approach to six experimentally synthesized DACs, with Fe as the first metal, and Mn,^43–45^ Co,^46–50^ Ni,^51–53^ Cu,^54–56^ Zn,^57,58^ and Pt^59^ as the second metal. This resulted in a total of 186 structures (31 configurations multiplied by 6 chemical compositions). By computing the formation energy, we found that the majority of these DACs are thermodynamically more stable than or at least comparable to their corresponding SACs (Figure 1b). This is consistent with the existing literature, which suggests that introducing a second metal site can enhance the stability of SACs. The calculated formation energy of the 186 DACs, along with their relative stability compared to that of the SACs, is listed in Table S1.

**Figure 1:**
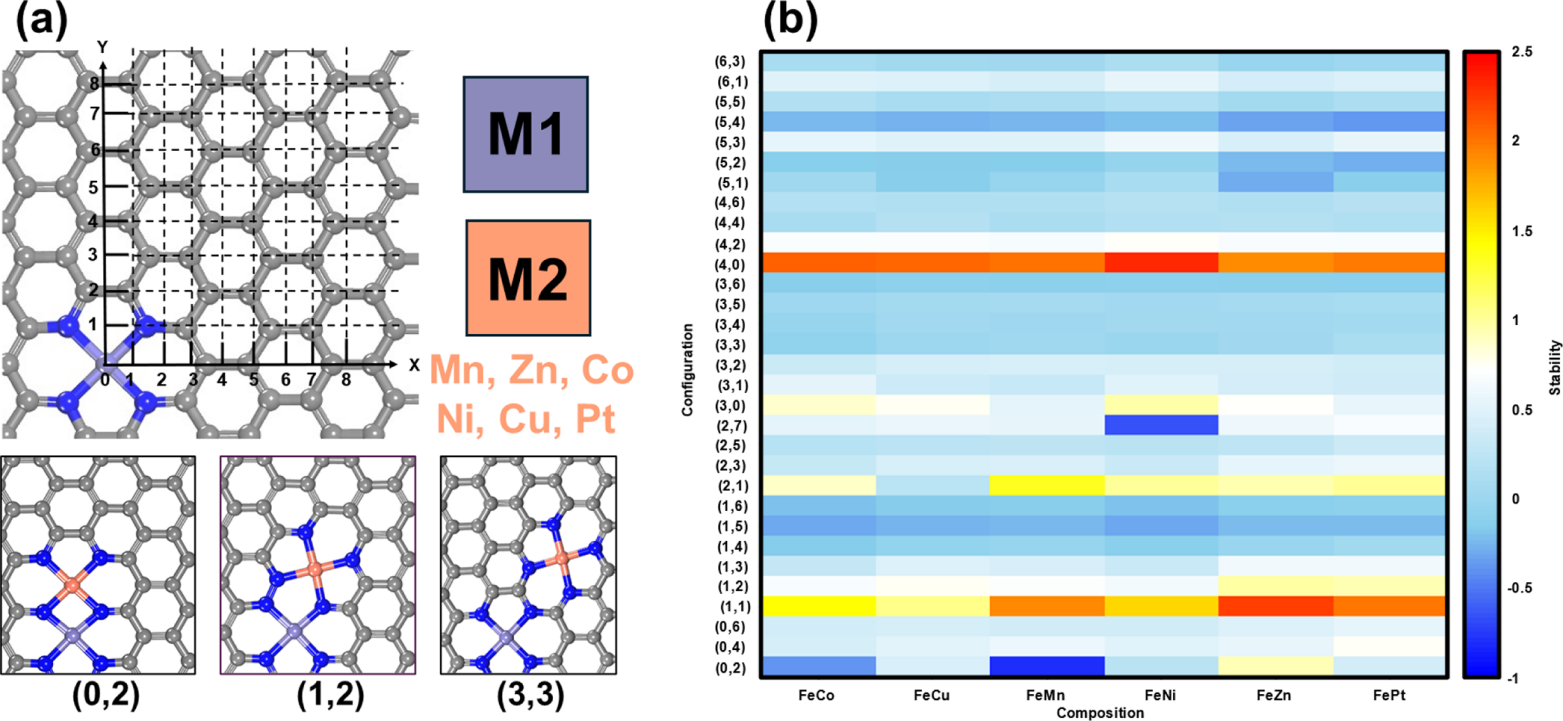
(a) Generation of the structural configuration for DACs by tiling the hexagonal lattice of graphene. C, gray; N, blue; Fe, purple; M, orange. (b) Relative stabilities of DACs compared to their corresponding SACs.

To investigate the catalyst’s structure under reaction conditions, we constructed voltage-dependent *ab initio* thermodynamic phase diagrams, accounting for all possible structures from the bare phase to the 2 ML OH phase. Figure S2 shows the atomic structures of the 9 phases considered. For each structure, we calculated the Gibbs free energy of formation as a function of potential, identifying the structure with the lowest energy at each potential as the most stable phase. We found that the atomic structure under reaction conditions of FeM–N–C DACs is primarily governed by the reactivity (i.e., standard reduction potential) of the second metal M. When it is less reactive than Fe with a more positive standard reduction potential, such as Pt, Cu, and Ni, the bare structure is the most stable at low potentials. However, as the potential increases, such as in the case of FeCu (0, 6) shown in Figure 3, Fe begins to oxidize, forming an *OH group starting at 0.29 V (vs RHE). As the potential continues to increase to 0.73 V (vs RHE), Fe undergoes further oxidation, with a second *OH group formed. Similar phenomena were observed for the majority of other FeCu–N–C, FeNi–N–C, and FePt–N–C DACs as summarized in Table S1.

We then examined the predicted structures under reaction conditions to investigate the ORR mechanism more thoroughly. To do this, we computed the free energy diagrams for the ORR along various pathways, including both the associative and dissociative pathways,^4,60^ at various sites (computational details are provided in the Supporting Information). We found that the reaction predominantly follows the associative pathway of the FeM–N–C DACs. Figure 3 shows the calculated ORR free energy diagram for the FeCu (0, 6) DAC. The reduction of *OH was determined to be the potential-limiting step, with the limiting potential predicted to be 0.73 V (vs RHE). Additionally, we found that the true active site is the OH-ligated Fe, rather than the bare Fe site. The Cu atom, being less reactive, primarily acts as a spectator, facilitating the reaction without directly interacting with the reaction intermediates. To investigate the specific role of Cu, we analyzed the projected density of states (PDOS) for FeCu–N–C (0, 6) DAC and Fe–N–C SAC. We found that the presence of the Cu moiety modulates the electronic structure of the Fe center, resulting in a distinct PDOS profile for FeCu–N–C (0, 6) DAC compared with Fe–N–C SAC (Figure S3). Specifically, the high ORR activity arises from significant Bader charge transfer and elevated spin density at the Fe site in FeCu–N–C (0, 6) DAC, which together weaken *OH adsorption and promote its desorption as H_2_O, as demonstrated in our previous work.^40^ This trend was observed for the majority of the FeCu–N–C, FeNi–N–C, and FePt–N–C DACs, as summarized in Table S1.

While this work focuses on thermodynamic analysis using free energy changes to identify the potential-limiting step, we acknowledge that a more comprehensive understanding of the ORR mechanism would benefit from explicit calculations of reaction barriers. Such kinetic analysis could reveal additional insights into transition states, activation energies, and the true rate-limiting step under the operational conditions. Future work incorporating transition state searches and reaction pathway analyses will be important for further validating and refining the catalytic trends identified in this work. Also, the spin state (magnetic moment) used for each metal in this work is as follows: Mn (3.9 μ_B_), Co (1.7 μ_B_), Cu (1.7 μ_B_), Fe (2.8 μ_B_), Ni (0.0 μ_B_), Pt (0.0 μ_B_), and Zn (0.0 μ_B_). These values were selected based on their known stability and are consistent with those reported in previous literature. To evaluate the influence of spin state on catalytic behavior, we selected the FeCu–N–C (0,6) DAC as a representative example. As shown in Table S2, variations in the spin state of Fe lead to noticeable changes in the ORR free energy profile. These results underscore the importance of considering the spin state in computational studies and suggest that a more systematic investigation of spin-state effects on ORR activity is warranted.

It is important to emphasize the connection between Figures 2 and 3. We found that the limiting potential predicted from the free energy diagram can also be derived from the *ab initio* phase diagram. Specifically, the limiting potential of 0.73 V (vs RHE) for the FeCu (0, 6) DAC, shown in Figure 3, corresponds to the potential at which the Fe­(OH) phase intersects with the Fe­(OH)­2 phase in Figure 2. Previous studies have demonstrated that the Gibbs free energy of *OH adsorption (ΔG_OH_) can serve as a descriptor for the ORR activity of SACs and DACs due to the linear scaling relationships between the adsorption energies of the ORR reaction intermediates. Our work, however, suggests that ΔG_OH_ on the bare catalyst structure may not always be sufficient to predict ORR activity (Figure 6). Instead, ΔG_OH_ under the reaction conditions should be considered, which can be directly obtained from the last intersecting potential of two stable phases in the *ab initio* phase diagrams. We will discuss this point in more detail later.

**Figure 2:**
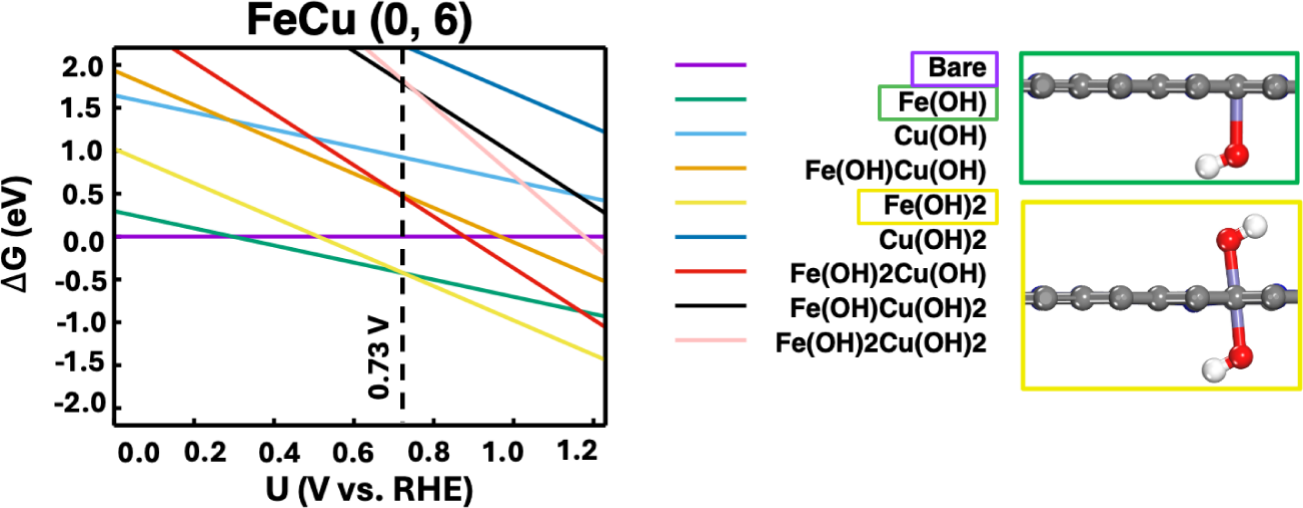
Voltage-dependent *ab initio* phase diagram for the FeCu (0, 6) DAC, along with the optimized atomic structures of the Fe­(OH) and Fe­(OH)_2_ phases. H, white; O, red; C, gray; Fe, purple.

**Figure 3:**
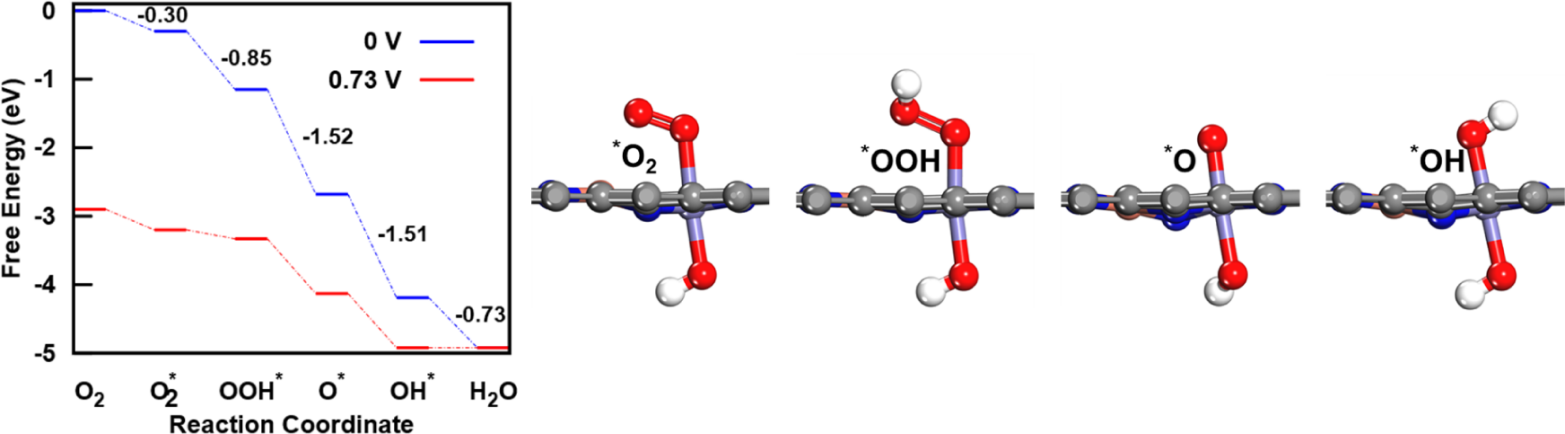
Free energy diagram of ORR along the associative pathway for FeCu (0, 6) DAC. Shown on the right are the optimized atomic structures for *O_2_, *OOH, *O, and *OH adsorption at the active site. C, gray; N, blue; Fe, purple; Cu, orange; O, red; and H, white.

For FeM–N–C DACs in which the second metal is too reactive and has a more negative standard reduction potential than Fe, such as Mn and Zn, we found that they also behave as spectators during the ORR. Figure 4 presents the *ab initio* phase diagram for the FeMn (0, 6) DAC. At a low potential of 0.22 V (vs RHE), an *OH group is adsorbed on the Mn site, followed by another *OH adsorption on the Fe site at 0.30 V (vs RHE). At a higher potential of 0.46 V (vs RHE), the Mn site is fully occupied by two *OH groups (structure outlined in black), leaving only the Fe site available for the ORR. The limiting potential for this structure was found to be 0.74 V (vs RHE), marked by the black dotted line in the phase diagram. We also performed calculations using the HSE06 hybrid functional to validate the *ab initio* phase diagram. As shown in Figure S4 and Table S3, the GGA-PBE functional generally overestimates *OH adsorption compared with HSE06, consistent with previous computational studies. As a result, the predicted limiting potential for the FeMn–N–C (0, 6) DAC increases from 0.74 to 0.81 V (vs RHE). Nevertheless, the relative stability of the various phases, and our conclusion regarding the active structure under reaction conditions, remains consistent between the GGA-PBE and HSE06 results. As summarized in Table S1, early-onset passivation of Mn was observed in most FeMn–N–C DACs, leading to OH-ligated Fe being identified as the true active site. Due to Zn’s relatively lower reactivity compared to Mn, we found that Zn is not fully passivated under reaction conditions, with the active site still being the OH-ligated Fe. Overall, our computational predictions are consistent with previous studies, which suggest that under reaction conditions, Zn and Mn in FeM–N–C DACs act as hydroxylated spectators, while Fe remains the active site for the ORR.^61,62^

**Figure 4:**
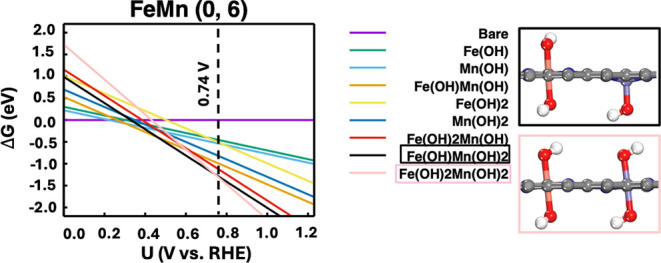
Voltage-dependent *ab initio* phase diagram for the FeMn (0, 6) DAC, along with the optimized atomic structures of the Fe­(OH)­Mn­(OH)_2_ and Fe­(OH)_2_Mn­(OH)_2_ phases. H, white; O, red; C, gray; Fe, purple; Mn, orange.

The situation becomes more complex when the reactivity of the second metal in FeM–N–C DACs is comparable to that of Fe. In this case, either Fe or the second metal (e.g., Co) can serve as the active site depending on the structural configuration. As shown in Figure 5, the active site is the bare Co, OH-ligated Co, and OH-ligated Fe for the (1, 4), (6, 3), and (3, 1) configurations of FeCo–N–C DACs, respectively. Table S1 lists the active sites for all 31 configurations. In summary, 19 configurations feature *OH adsorption exclusively on the Fe sites, with the bare Co sites acting as active sites. The remaining 12 configurations show *OH adsorption on both Fe and Co sites, with 9 configurations having OH-adsorbed Co as the active site and 3 configurations having OH-adsorbed Fe as the active site. Our computational predictions, which suggest that Co’s role as the active site is influenced by the dual-metal arrangement, are in good agreement with recent studies.^63^ While the bifunctional ORR/OER performance of these DACs is not discussed here, it would be an interesting direction for future exploration.

**Figure 5:**
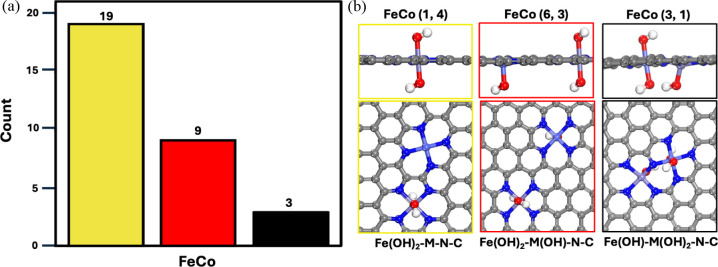
(a) Histogram of the active sites for the 31 configurations of FeCo–N–C DACs. Yellow corresponds to the Co active site, red to the OH-ligated Co active site, and black to the OH-ligated Fe active site. (b) The optimized atomic structures for the representative configurations with various active sites. H, white; O, red; C, gray; N, blue; Fe, purple; Co, light blue.

We examined in total 186 FeM–N–C DACs for the ORR, summarizing the formation energy, active site under reaction conditions, and limiting potentials for these catalysts in Table S1. A heat map of the limiting potentials is shown in Figure S5. To illustrate the impact of metal composition on active site distribution and the resulting structural diversity among the DACs, the number of each type of active site identified across all DACs is summarized in Table S4. Compared to the state-of-the-art ORR catalyst Pt, we found that 29 of the DACs outperform Pt, exhibiting a limiting potential greater than 0.8 V (vs RHE). Figure S6 shows the voltage-dependent *ab initio* phase diagrams for these 29 high-performing DACs. Notably, 19 of them are from the FeCo combination, while the remaining 10 include compositions from FeNi, FeMn, FeCu, FeZn, and FePt (Table S5). This computational prediction aligns well with experimental observations, where FeCo–N–C DACs have been frequently identified in the literature as highly effective ORR catalysts, as illustrated in Figure 6b.^25,64^ It is important to note that conventional computational methods, which do not account for dynamic structural evolution under reaction conditions, fail to capture these insights. As shown in Figure 6a, when directly using the bare catalyst structures, only two DACs were predicted to be active for the ORR, while the majority were predicted to be inactive, contradicting experimental findings.^65,66^

**Figure 6:**
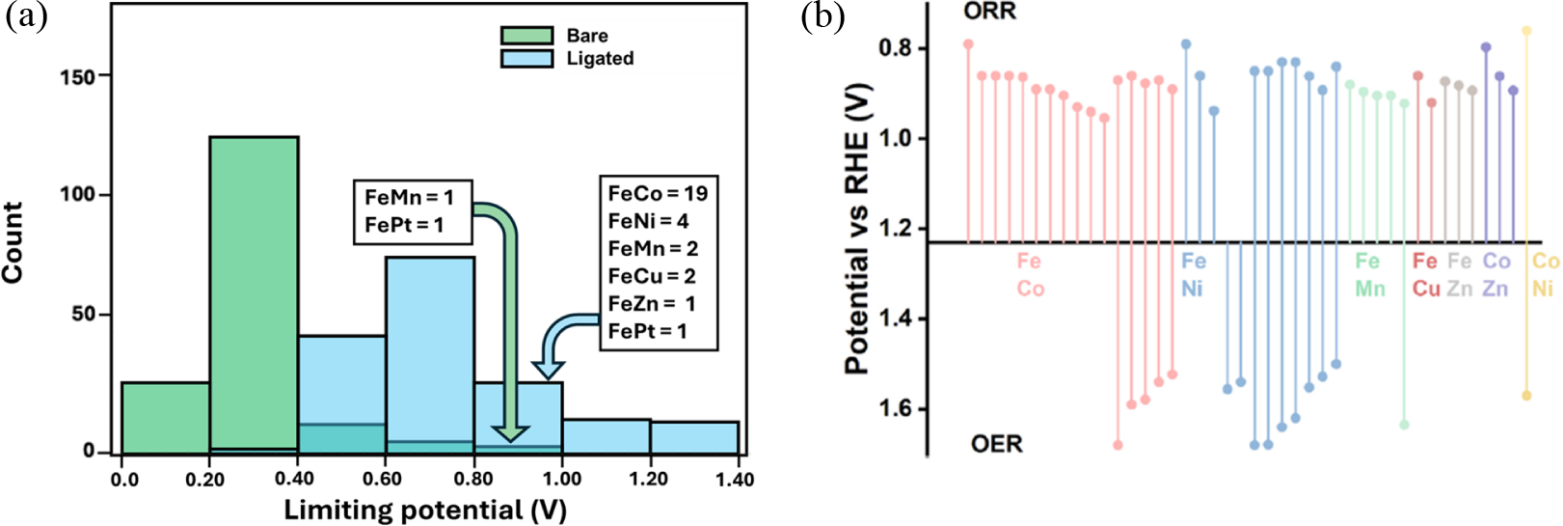
(a) Comparison of the predicted limiting potential for 186 DACs based on the catalyst’s bare structure (green) vs the structure under reaction conditions (blue). (b) Experimental ORR and OER performances of FeM–N–C DACs. Reprinted from *Nano Energy*, Vol. 104, Wang et al., *Dual-atom catalysts for oxygen electrocatalysis*, pp. 107927, Copyright (2022), with permission from Elsevier.

This discrepancy between computational predictions based on bare catalyst structures and experimental findings highlights the critical need to consider the electrochemically modified structures that form under working conditions as they can significantly impact catalytic performance. Our results underscore the importance of computational approaches that more accurately capture the active sites during electrocatalytic reactions, such as the *ab initio* phase diagram presented here, to provide deeper insights into catalytic mechanisms and facilitate the efficient prediction of novel catalysts. We included a figure illustrating the correlation between the ORR limiting potential and the last intersection potential derived from the *ab initio* phase diagram, supporting its use as an effective descriptor for ORR activity. As shown in Figure S7, for DACs located on the left side of the volcano plot, strong *OH binding under the reaction conditions makes *OH reduction the potential-limiting step. In contrast, DACs on the right side exhibit weak *OOH binding, making *OOH formation the potential-limiting step. We find the FeNi–N–C (0, 2) DAC is positioned at the peak of the volcano plot, exhibiting an optimal *OH binding energy under reaction conditions and an ORR limiting potential of 0.93 V (vs RHE).

## Conclusions

In this study, we performed detailed DFT calculations to explore the structural evolution of 186 FeM–N–C DACs under the reaction conditions. By constructing *ab initio* phase diagrams, we revealed how electrochemically modified metal sites emerge in response to the applied potential. Based on the reactivity of the second metal in FeM–N–C, we categorized them into three groups: less reactive, moderately reactive, and highly reactive. Our findings show that less reactive metals remain bare under working conditions, while highly reactive metals become fully passivated, leaving Fe as the sole active site. Moderately reactive metals, such as Co, can serve as either active sites or spectators, contributing to a range of possible reaction pathways. Moreover, by calculating the ORR limiting potential for the DACs using the predicted structures under the reaction conditions, we demonstrated that the ORR activity can be directly predicted from the phase diagrams. Our work offers valuable insights into the catalytic mechanisms of FeM–N–C DACs and presents an efficient method for predicting catalytic activity through *ab initio* phase diagrams. Additionally, the data generated here can be further used to train machine learning models, enabling the screening of a broader range of catalytic materials.

## Supplementary Material



## Notes

Detailed computational methods, atomic structures of the 31 configurations and 9 phases, PDOS comparison between DAC and SAC, heat map of predicted limiting potentials, *ab initio* phase diagrams for the top 29 DACs, complete DFT-calculated properties for all 186 DACs, analysis of spin effects on ORR activity, and comparison between the GGA-PBE and HSE06 functionals (PDF)
