# Supplementary material for: Dual-Atom Catalysts for the Oxygen Reduction Reaction: Unraveling Atomic Structures under Reaction Conditions
Source: J Am Chem Soc. 2025 May 23;147(22):19210–6. doi: 10.1021/jacs.5c04776 (PMC12147109; doi:10.1021/jacs.5c04776)
Supplement: Supplementary file 1 [file ja5c04776_si_001.pdf]

Online Supporting Information for

Dual atom catalysts for the oxygen reduction reaction: Unraveling atomic structures under reaction conditions

Courtney Brea[1,2], Guoxiang Hu[3,4,*]

[1]Department of Chemistry and Biochemistry, Queens College, City University of New York, New York, NY 11367, USA

[2]The Graduate Center, City University of New York, New York, NY 10016, USA

[3]School of Materials Science and Engineering, Georgia Institute of Technology, Atlanta, Georgia, 30332, USA

[4]School of Chemistry and Biochemistry, Georgia Institute of Technology, Atlanta, Georgia 30332, USA

*Corresponding author. Email: emma.hu@mse.gatech.edu

COMPUTATIONAL METHODS

1. Computation details

Spin-polarized DFT calculations were performed using the Vienna *ab initio* simulation package (VASP).[1,2] Electron exchange-correlation was represented by the functional of Perdew, Burke and Ernzerhof (PBE) of generalized gradient approximation (GGA).[3] The ion-electron interaction was described with the projector augmented wave (PAW) method.[4] The plane-wave cutoff was set to 400 eV, and a conjugate gradient method was applied to relax the geometry until interatomic forces are less than 0.025 eV/Å.

The FeM-N-C (M = Co, Cu, Mn, Ni, Pt, Zn) dual atom catalysts were modeled by FeM-N_x clusters (x = 6, 7, or 8) embedded in 6 × 7 graphene supercells. The perfect graphene monolayer contains 84 carbon atoms with cell dimensions of a = 17.22 Å and b = 14.76 Å. A vacuum of 20 Å along the *z*-direction was applied for the two-dimensional graphene monolayer, which is periodic in the *xy*-plane, to avoid unphysical interactions between periodic images. The Brillouin zone was sampled by (3 × 3 × 1) Monkhorst–Pack *k*-point mesh.

2. Formation energy

To evaluate the thermodynamic stability of each DAC, we computed the formation energy E_f using $E_{f(FeM)} = E_{FeM} - (E_{graphene} - y\mu_C + x\mu_N + \mu_M + \mu_{Fe})$.[5] E_{FeM} is the total energy of the FeM-N-C DAC. $E_{graphene}$ is the total energy of the perfect graphene. μ_C is the chemical potential of carbon atoms defined as the atomic energy of perfect graphene,[6,7] and y is the number of carbon atoms removed from the perfect graphene. μ_N is the chemical potential of nitrogen atoms defined as half the total energy of an N_2 molecule,[7] and x the number of nitrogen atoms included in the dual-metal center.

μ_M and μ_{Fe} are the chemical potentials of the second metal and iron in the gas phase. To compare the formation energy of DACs with SACs, we compute the formation energy of SACs by using $E_{f(M)} = E_M – (E_{graphene} - y\mu_C + x\mu_N + \mu_M)$ and $E_{f(Fe)} = E_M – (E_{graphene} - y\mu_C + x\mu_N + \mu_{Fe})$. We take the difference between the formation energy of DACs and their respective SACs by using $E_{f(Fe-M)} – (E_{f(Fe)} + E_{f(M)})$. The differences were plotted in Figure 1b.

3. *ab initio* thermodynamic phase diagrams

To understand the atomic structures of the DACs under reaction conditions, we construct the phase diagrams for all 186 structures using the concept of the theoretical standard hydrogen electrode (SHE).[8] It is assumed the surface is in equilibrium with protons and liquid water at 298 K, so that hydroxyl groups may be exchanged between the surface and a reference electrolyte through the steps of $H_2O(l) + * \leftrightarrow HO^* + H^+(aq) + e^-$. The oxidation of water to *OH depends on the applied potential through the chemical potential of the proton electron pair. The reaction $H^+(aq) + e^- \leftrightarrow 1/2H_2(g)$ is defined to be in equilibrium at zero voltage, and the chemical potential of the proton electron pair can be calculated by $1/2\ G_{H2}$ - eU. We consider the following surface structures of FeM-N-C DACs: 1/2 ML Fe(OH), 1/2 ML M(OH), 1 ML OH, 1 ML Fe(OH), 1 ML MOH, 3/2 ML Fe(OH), 3/2 ML M(OH), and 2 ML OH, and the bare surface. From this method, the free energy of a given surface structure can be calculated as a function of the applied potential by using

$$\Delta G\ (1/2\ ML\ Fe(OH)) = (G_{*Fe(OH)} + 1/2G_{H2} – eU) – G_{Fe\text{-}M} – G_{H2O}$$

$$\Delta G\ (1/2\ ML\ M(OH)) = (G_{*M(OH)} + 1/2G_{H2} – eU) – G_{Fe\text{-}M} – G_{H2O}$$

$$\Delta G\ (1\ ML\ OH) = (G_{*2OH} + G_{H2} – 2eU) – G_{Fe\text{-}M} – 2G_{H2O}$$

$$\Delta G\ (1\ ML\ Fe(OH)) = (G_{*Fe(2OH)} + G_{H2} - 2eU) - G_{Fe\text{-}M} - 2G_{H2O}$$

$$\Delta G\ (1\ ML\ M(OH)) = (G_{*M(2OH)} + G_{H2} - 2eU) - G_{Fe\text{-}M} - 2G_{H2O}$$

$$\Delta G\ (3/2\ ML\ Fe(OH)) = (G_{*Fe(3OH)} + 3/2G_{H2} - 3eU) - G_{Fe\text{-}M} - 3G_{H2O}$$

$$\Delta G\ (3/2\ ML\ M(OH)) = (G_{*M(3OH)} + 3/2G_{H2} - 3eU) - G_{Fe\text{-}M} - 3G_{H2O}$$

$$\Delta G\ (2\ ML\ OH) = (G_{*4OH} + 2G_{H2} - 4eU) - G_{Fe\text{-}M} - 4G_{H2O}$$

where $G_{Fe\text{-}M}$ is the free energy of the bare FeM-N-C DAC. $G_{*Fe(OH)}$, $G_{*M(OH)}$, G_{*2OH}, $G_{*Fe(2OH)}$, $G_{*M(2OH)}$, $G_{*Fe(3OH)}$, $G_{*M(3OH)}$ and G_{*4OH} is the free energy of the catalyst covered by one *OH, two *OH, three *OH, and four *OH either at the M site, Fe site, or both, respectively. G_{H2O} and G_{H2} are the free energies of H_2O and H_2 molecules.

4. Oxygen reduction reaction activity

We compute the free energy diagrams for FeM-N-C DACs by using the computational hydrogen electrode.[9,10] The change in Gibbs free energy for each elementary step can be calculated as

$$\Delta G = \Delta E + \Delta ZPE - T\Delta S + \Delta G_U$$

where ΔE, ΔZPE, and ΔS is the change in the total energy, zero-point energy, and entropy, respectively. The temperature (T) was assumed to be 298.15 K. $\Delta G_U = -neU$, where n and U are the number of transferred electrons for each step and the electrode potential, respectively. In this work, we assume full cancellation of zero-point energy, entropy, and solvation effect corrections,

and report pathways based solely on calculated binding energies from internal energies. This cancellation was previously validated for ORR intermediates on Pt surfaces.[11–13]

The detailed reaction pathway for the 4e$^-$ association mechanism is as follows

$$O_2 + H^+ + e^- \rightarrow {}^*OOH \text{ (R1)}$$

$$^*OOH + H^+ + e^- \rightarrow *O + H_2O \text{ (R2)}$$

$$*O + H^+ + e^- \rightarrow *OH \text{ (R3)}$$

$$*OH + H^+ + e^- \rightarrow * + H_2O \text{ (R4)}$$

The reaction free energy for each step can be calculated using

$$\Delta G_1 = G_{*OOH} - G_{O2} - 0.5G_{H2} + eU$$

$$\Delta G_2 = G_{*O} + G_{H2O} - G_{*OOH} - 0.5G_{H2} + eU$$

$$\Delta G_3 = G_{*OH} - G_{*O} - 0.5G_{H2} + eU$$

$$\Delta G_4 = G_{H2O} + G_{*} - G_{*OH} - 0.5G_{H2} + eU$$

Since DFT is struggling with calculating the triplet ground state of the O_2 molecule, we calculate the reaction free energy in its place using the following

$$*+ 2H_2O \leftrightarrow *OOH + 3/2H_2$$

$$*+H_2O \leftrightarrow *O + H_2$$

$$*+H_2O \leftrightarrow *OH + 1/2H_2$$

In this way, the reaction free energy can be defined as

$$\Delta G_{*OOH} = 1.5G_{H2} + G_{*OOH} - 2G_{H2O} - G_{*}$$

$$\Delta G_{*O} = G_{H2} + G_{*O} - G_{H2O} - G_{*}$$

$$\Delta G_{*OH} = 0.5G_{H2} + G_{*OH} - G_{H2O} - G_{*}$$

At the equilibrium potential, 1.23V, the reaction free energy of $O_2 + 4e^- \leftrightarrow 2H_2O$ is 4.92eV. From here, $\Delta G_1 = -4.92 - (\Delta G_2 + \Delta G_3 + \Delta G_4)$. The reaction free energy of the four-electron process is represented as

$$\Delta G_1 = \Delta G_{*OOH} - 4.92 + eU$$

$$\Delta G_2 = \Delta G_{*O} - \Delta G_{*OOH} + eU$$

$$\Delta G_3 = \Delta G_{*OH} - \Delta G_{*O} + eU$$

$$\Delta G_4 = \Delta G_{*OH} + eU$$

Therefore, the reaction free energy of each elementary step can be expressed by the adsorption free energies of ΔG_{*OOH}, ΔG_{*O}, and ΔG_{*OH}. Moreover, we divide R1 into two steps to account for physisorption of O_2 at the surface as (R1') $O_2 \rightarrow {}^*O_2$; (R1") ${}^*O_2 + H^+ + e^- \rightarrow {}^*OOH$. Hence, the adsorption free energy of O_2 can be calculated as $\Delta G_1' = \Delta G_1 - \Delta G_1''$. Using the calculated reaction free energies, the ORR free energy diagrams were plotted. The elementary step with the least negative change in free energy is the thermodynamic limiting step. Since ΔG is calculated at U = 0 V, we define the limiting potential as $U_L = -\frac{\max(\Delta G_1, \Delta G_2, \Delta G_3, \Delta G_4)}{e}$.

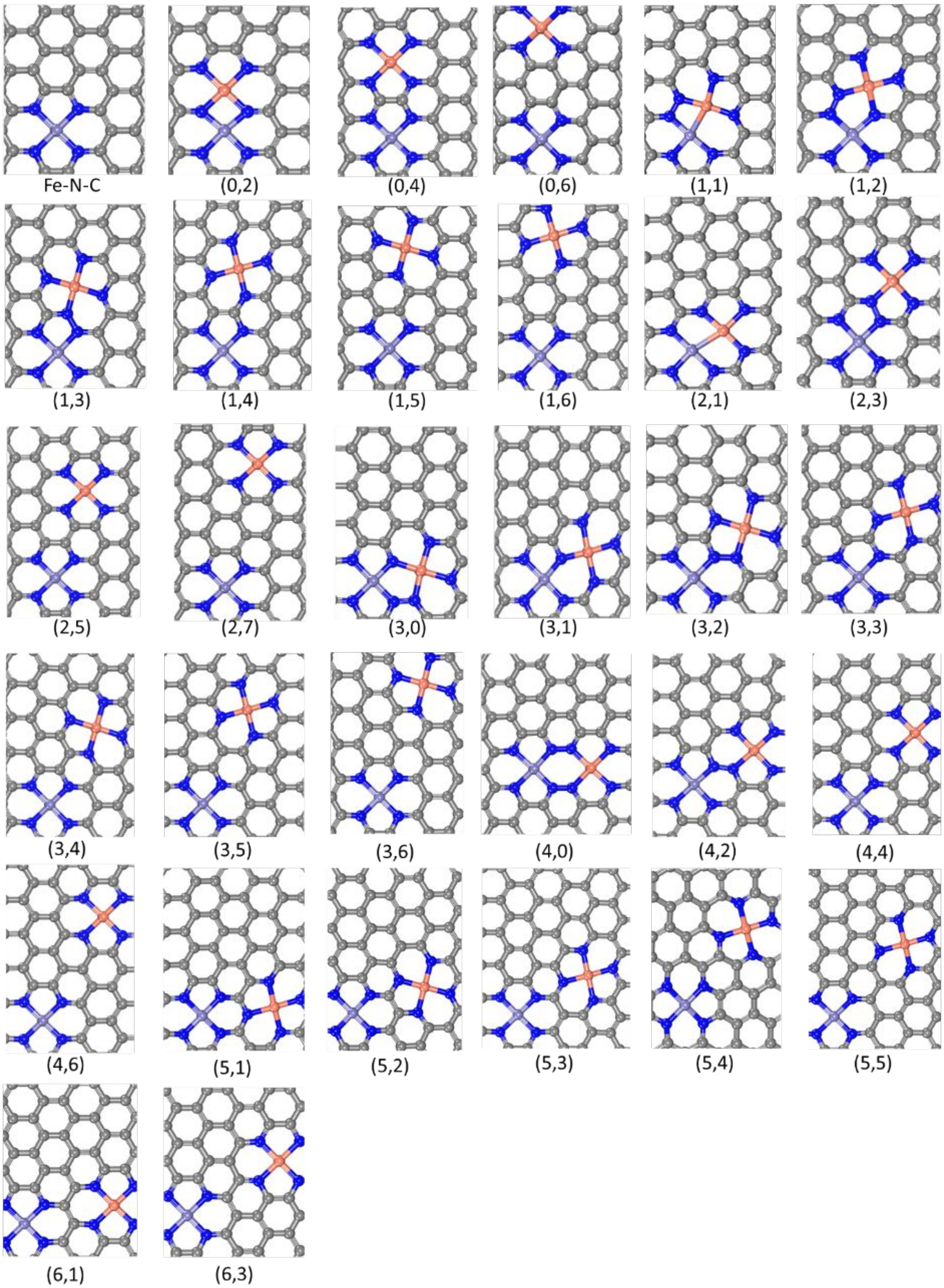

Figure S1. Atomic structures for the 31 configurations of FeM-N-C DACs. The atomic structure of the Fe-N-C SAC is also shown. Fe, taupe; M, orange; N, blue; C, grey.

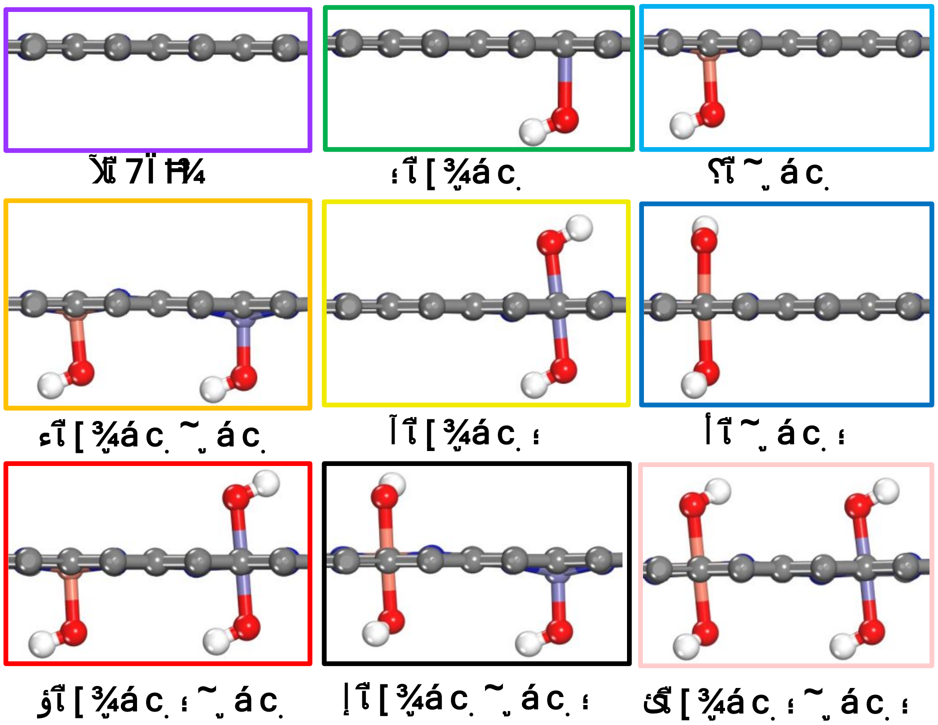

Figure S2. Atomic structures for the bare, Fe(OH), M(OH), Fe(OH)M(OH), Fe(OH)2, M(OH)2, Fe(OH)2M(OH), Fe(OH)M(OH)2, and Fe(OH)2M(OH)2 phases of DACs. Fe, taupe; M, orange; N, blue; C, grey. O, red; H, white.

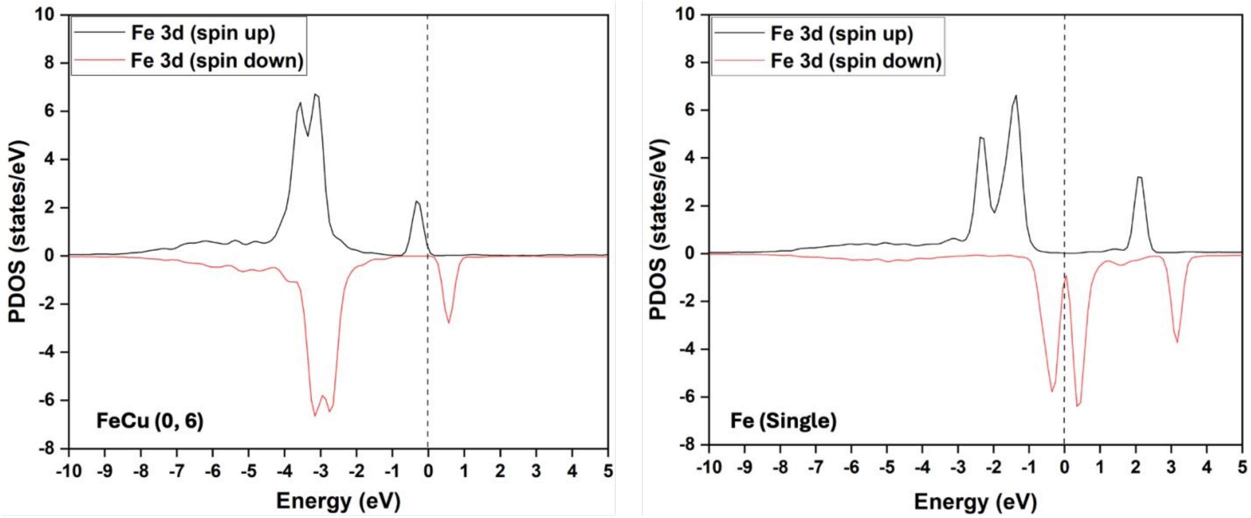

Figure S3. The projected density of states for FeCu-N-C (0, 6) DAC and Fe-N-C SAC. The Fermi level is set as zero.

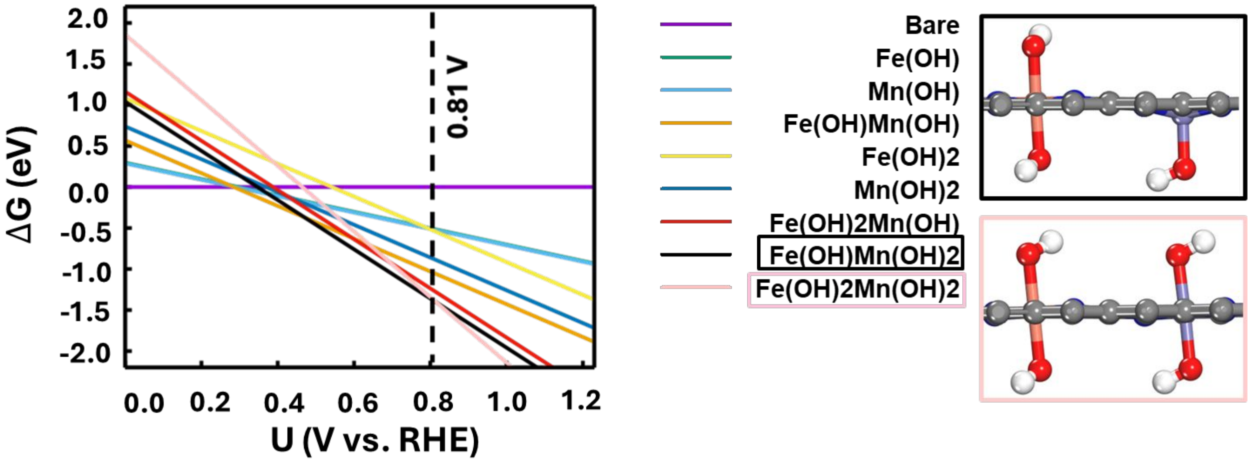

Figure S4. Voltage-dependent *ab initio* phase diagram for the FeMn-N-C (0, 6) DAC calculated using the HSE06 functional, along with the optimized atomic structures of the Fe(OH)Mn(OH)2 and Fe(OH)2Mn(OH)2 phases. H, white; O, red; C, grey; Fe, purple; Mn, orange.

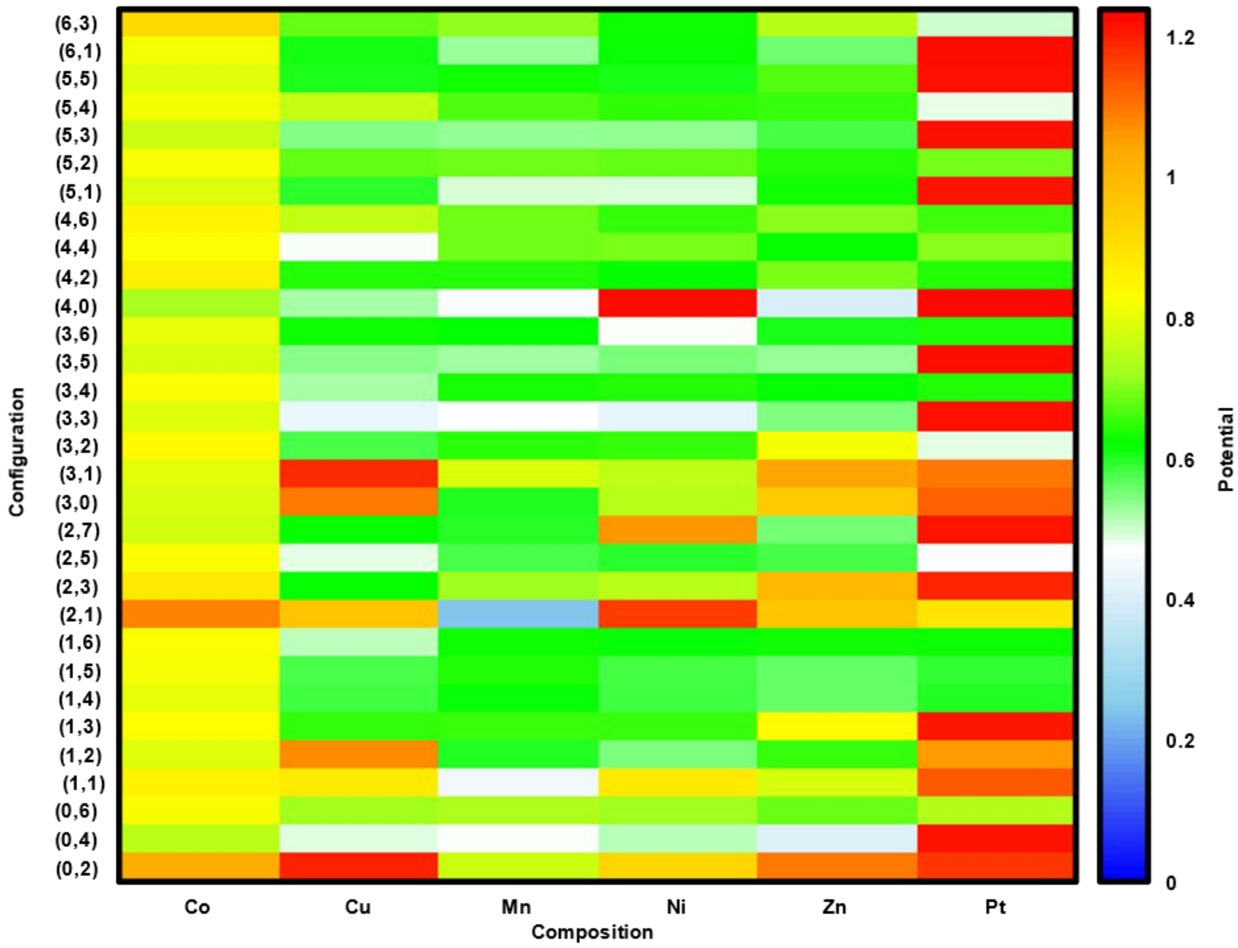

Figure S5. Heat map of the predicted limiting potentials for all 186 FeM-N-C (M = Co, Cu, Mn, Ni, Zn, and Pt) DACs.

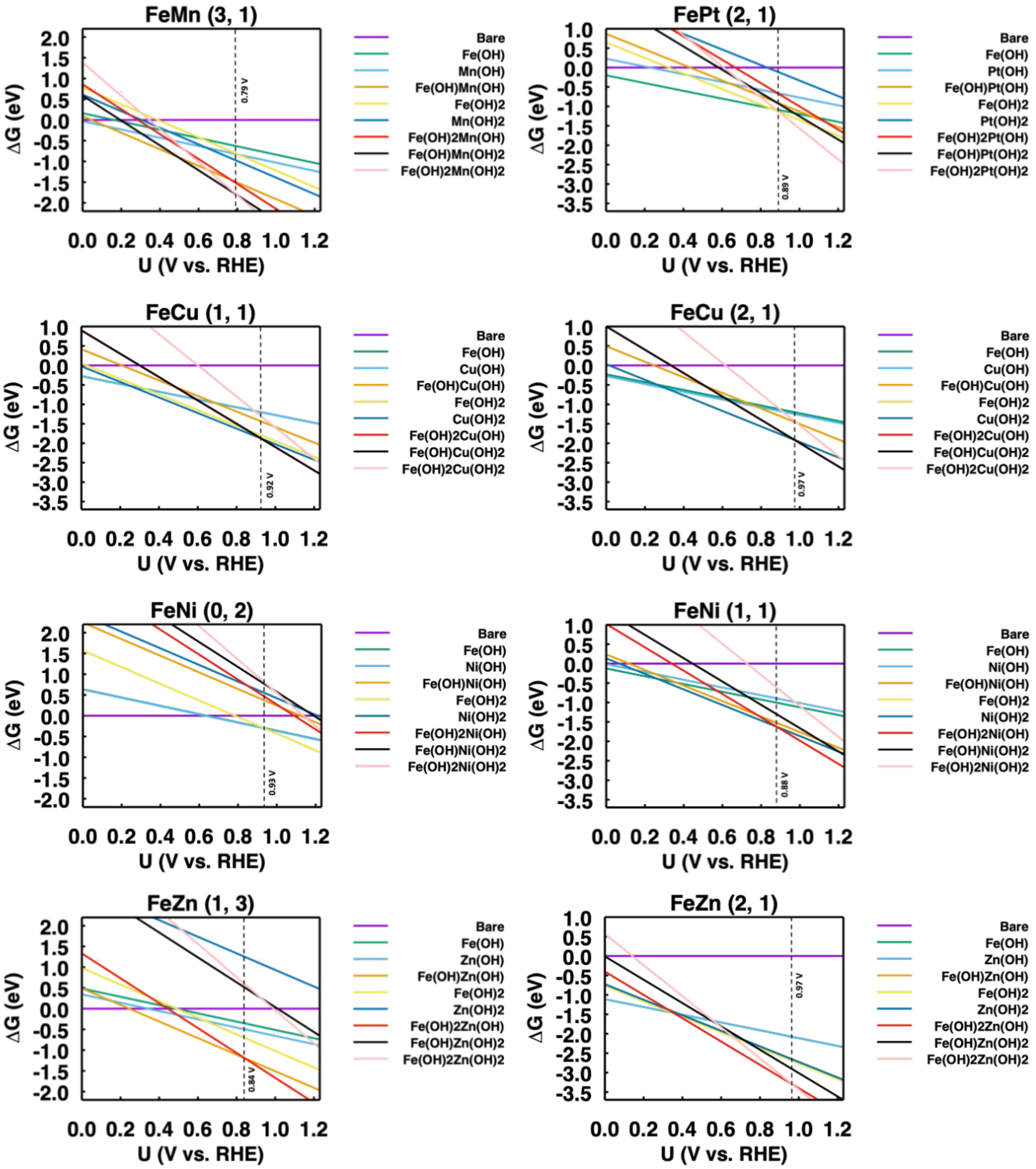

FeMn (3, 1)
ΔG (eV)
U (V vs. RHE)
0.79 V
Bare
Fe(OH)
Mn(OH)
Fe(OH)Mn(OH)
Fe(OH)2
Mn(OH)2
Fe(OH)2Mn(OH)
Fe(OH)Mn(OH)2
Fe(OH)2Mn(OH)2
FePt (2, 1)
ΔG (eV)
U (V vs. RHE)
0.89 V
Bare
Fe(OH)
Pt(OH)
Fe(OH)Pt(OH)
Fe(OH)2
Pt(OH)2
Fe(OH)2Pt(OH)
Fe(OH)Pt(OH)2
Fe(OH)2Pt(OH)2
FeCu (1, 1)
ΔG (eV)
U (V vs. RHE)
0.92 V
Bare
Fe(OH)
Cu(OH)
Fe(OH)Cu(OH)
Fe(OH)2
Cu(OH)2
Fe(OH)2Cu(OH)
Fe(OH)Cu(OH)2
Fe(OH)2Cu(OH)2
FeCu (2, 1)
ΔG (eV)
U (V vs. RHE)
0.97 V
Bare
Fe(OH)
Cu(OH)
Fe(OH)Cu(OH)
Fe(OH)2
Cu(OH)2
Fe(OH)2Cu(OH)
Fe(OH)Cu(OH)2
Fe(OH)2Cu(OH)2
FeNi (0, 2)
ΔG (eV)
U (V vs. RHE)
0.93 V
Bare
Fe(OH)
Ni(OH)
Fe(OH)Ni(OH)
Fe(OH)2
Ni(OH)2
Fe(OH)2Ni(OH)
Fe(OH)Ni(OH)2
Fe(OH)2Ni(OH)2
FeNi (1, 1)
ΔG (eV)
U (V vs. RHE)
0.88 V
Bare
Fe(OH)
Ni(OH)
Fe(OH)Ni(OH)
Fe(OH)2
Ni(OH)2
Fe(OH)2Ni(OH)
Fe(OH)Ni(OH)2
Fe(OH)2Ni(OH)2
FeZn (1, 3)
ΔG (eV)
U (V vs. RHE)
0.84 V
Bare
Fe(OH)
Zn(OH)
Fe(OH)Zn(OH)
Fe(OH)2
Zn(OH)2
Fe(OH)2Zn(OH)
Fe(OH)Zn(OH)2
Fe(OH)2Zn(OH)2
FeZn (2, 1)
ΔG (eV)
U (V vs. RHE)
0.97 V
Bare
Fe(OH)
Zn(OH)
Fe(OH)Zn(OH)
Fe(OH)2
Zn(OH)2
Fe(OH)2Zn(OH)
Fe(OH)Zn(OH)2
Fe(OH)2Zn(OH)2

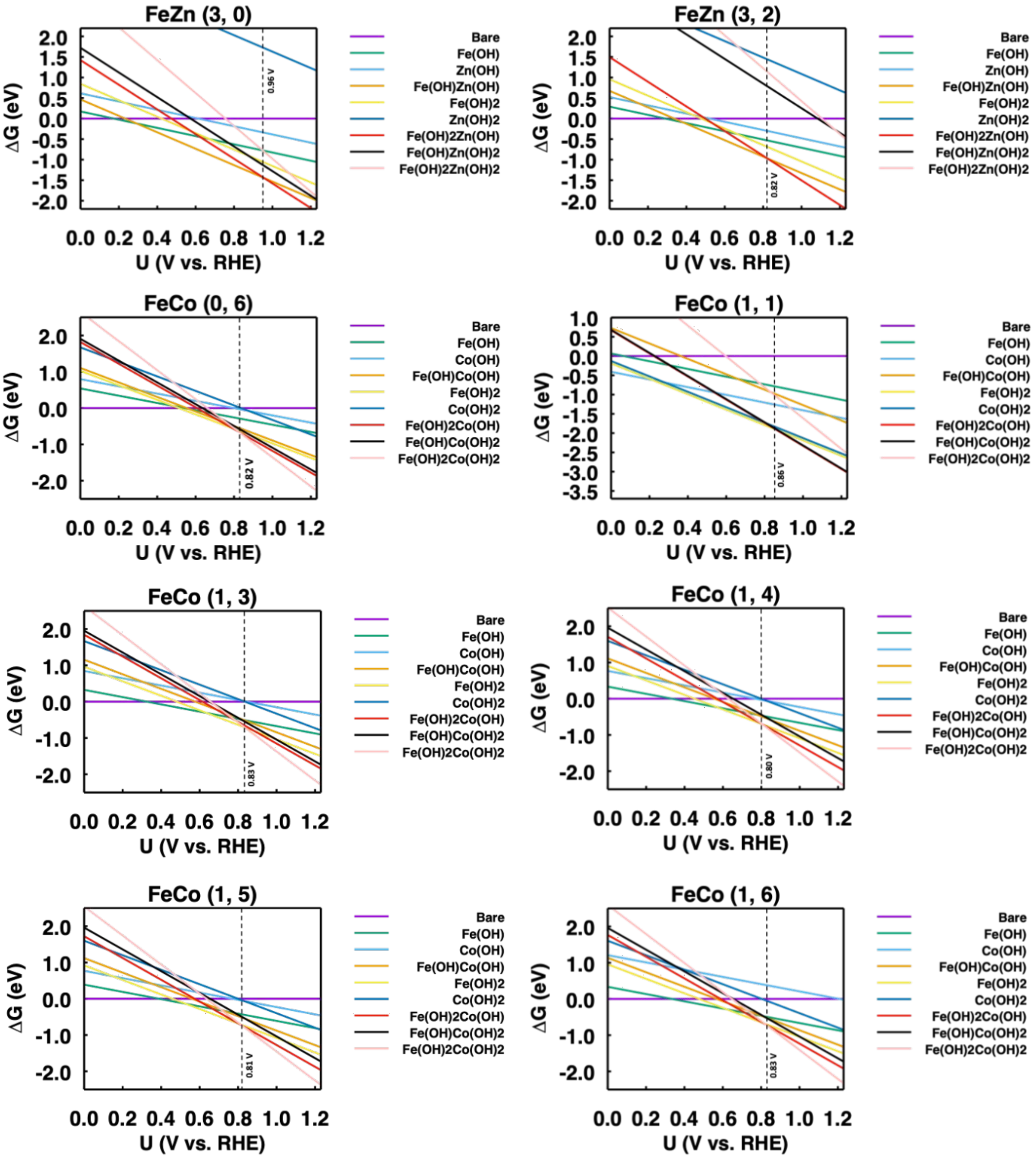
FeZn (3, 0)
0.96 V
FeZn (3, 2)
0.82 V
Bare
Fe(OH)
Zn(OH)
Fe(OH)Zn(OH)
Fe(OH)2
Zn(OH)2
Fe(OH)2Zn(OH)
Fe(OH)Zn(OH)2
Fe(OH)2Zn(OH)2
FeCo (0, 6)
0.82 V
FeCo (1, 1)
0.86 V
FeCo (1, 3)
0.83 V
FeCo (1, 4)
0.80 V
FeCo (1, 5)
0.81 V
FeCo (1, 6)
0.83 V
Bare
Fe(OH)
Co(OH)
Fe(OH)Co(OH)
Fe(OH)2
Co(OH)2
Fe(OH)2Co(OH)
Fe(OH)Co(OH)2
Fe(OH)2Co(OH)2
ΔG (eV)
U (V vs. RHE)

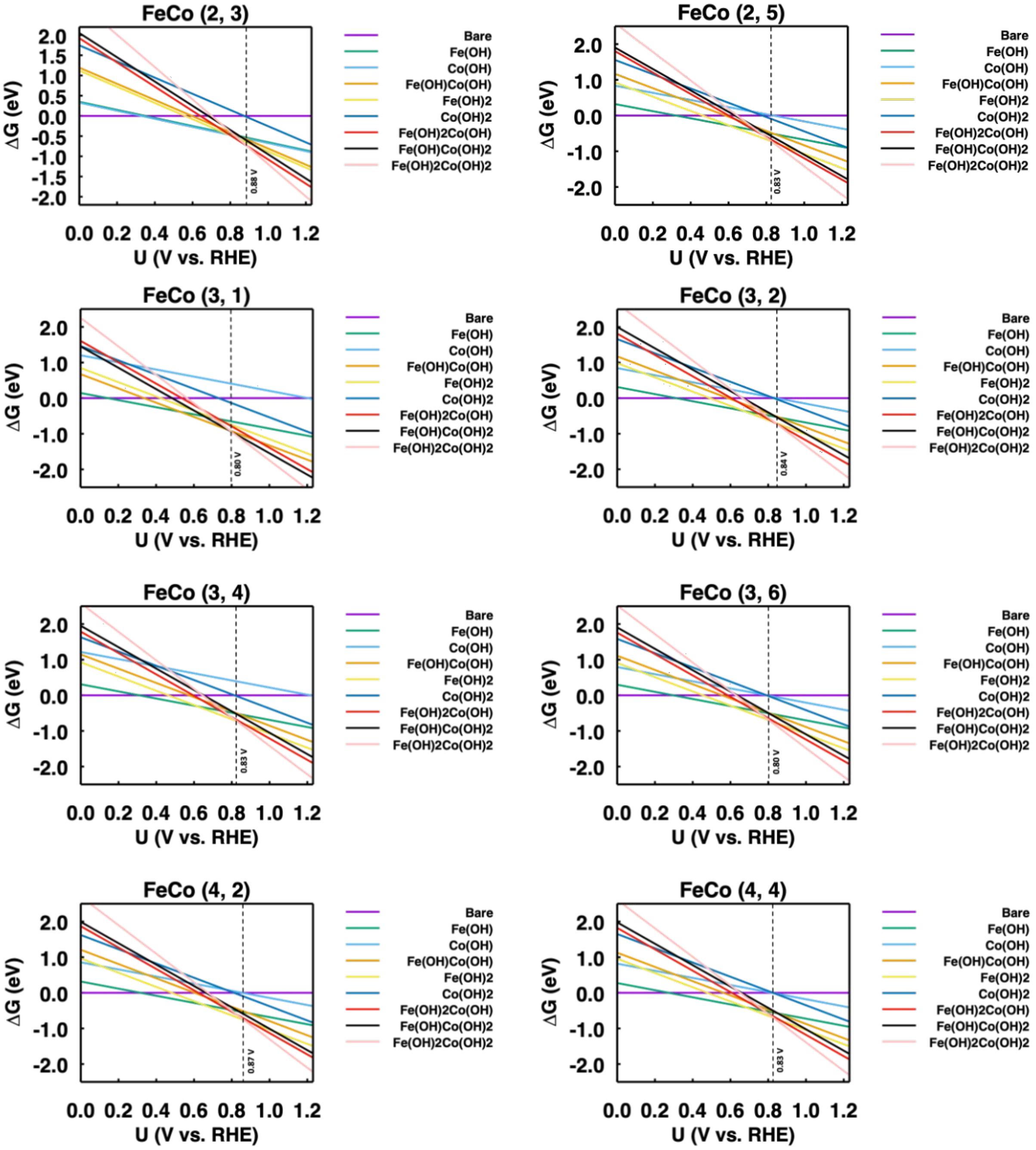
FeCo (2, 3)
ΔG (eV)
U (V vs. RHE)
0.88 V
Bare
Fe(OH)
Co(OH)
Fe(OH)Co(OH)
Fe(OH)2
Co(OH)2
Fe(OH)2Co(OH)
Fe(OH)Co(OH)2
Fe(OH)2Co(OH)2
FeCo (2, 5)
0.83 V
FeCo (3, 1)
0.80 V
FeCo (3, 2)
0.84 V
FeCo (3, 4)
0.83 V
FeCo (3, 6)
0.80 V
FeCo (4, 2)
0.87 V
FeCo (4, 4)
0.83 V

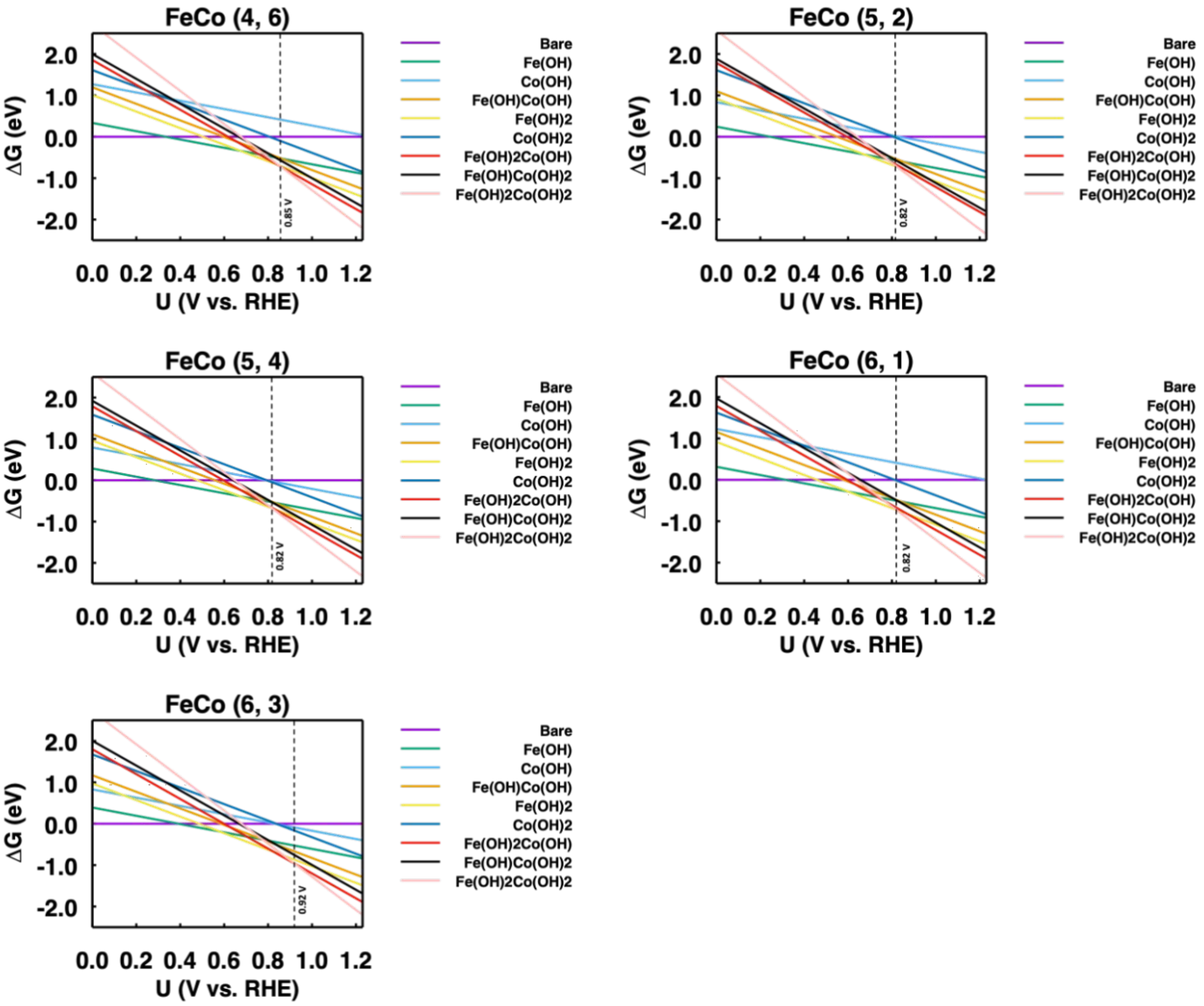

Figure S6. Voltage-dependent *ab initio* phase diagrams for the 29 DACs that outperform Pt.

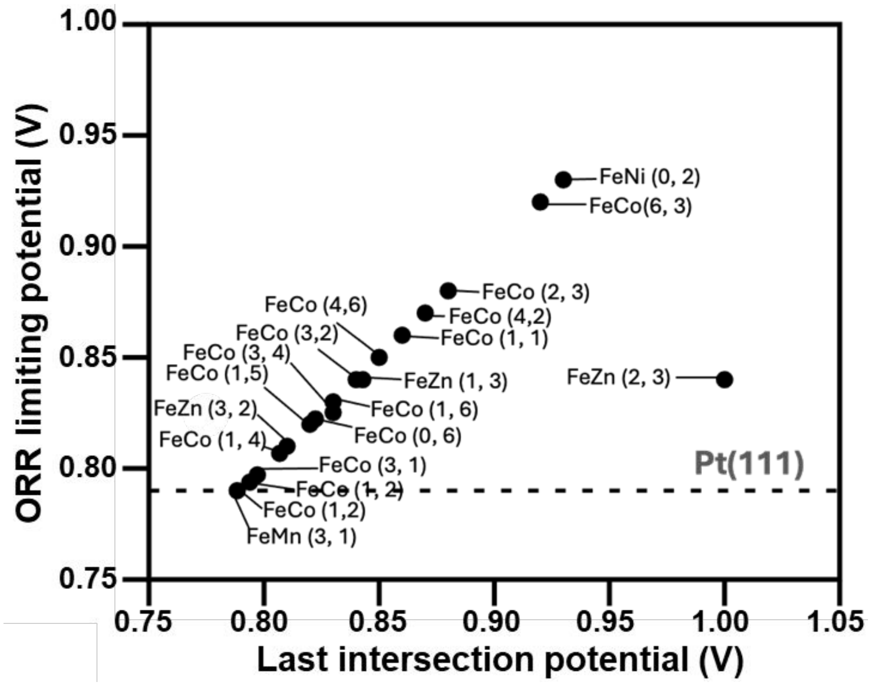

Figure S7. Correlation between the ORR limiting potential and the last intersection potential derived from the *ab initio* phase diagram, supporting its use as an effective descriptor for ORR activity.

Table S1. A summary of formation energies, relative stabilities, active site under reaction conditions, and limiting potentials for all 186 DACs.

DAC	Formation Energy (eV)	Relative Stability (eV)	Active Site	Limiting Potential (V)
**FeCo:(0,2)**	2.030	-0.400	Fe(OH)2	1.03
**FeCo:(0,4)**	2.836	0.406	Fe(OH)2	0.75
**FeCo:(0,6)**	2.821	0.392	Fe(OH)2Co(OH)	0.82
**FeCo:(1,1)**	3.859	1.429	Fe(OH)2	0.86
**FeCo:(1,2)**	3.092	0.663	Fe(OH)2Co(OH)	0.79
**FeCo:(1,3)**	2.719	0.290	Fe(OH)2	0.83
**FeCo:(1,4)**	2.251	-0.178	Fe(OH)2Co(OH)	0.80
**FeCo:(1,5)**	2.109	-0.321	Fe(OH)2Co(OH)	0.81
**FeCo:(1,6)**	2.207	-0.222	Fe(OH)2Co(OH)	0.83
**FeCo:(2,1)**	3.303	0.874	Fe(OH)Co(OH)2	1.09
**FeCo:(2,3)**	2.724	0.294	Fe(OH)2Co(OH)	0.88
**FeCo:(2,5)**	2.616	0.187	Fe(OH)2	0.83
**FeCo:(2,7)**	2.947	0.518	Fe(OH)2	0.78
**FeCo:(3,0)**	3.287	0.857	Fe(OH)Co(OH)2	0.78
**FeCo:(3,1)**	2.957	0.528	Fe(OH)Co(OH)2	0.80
**FeCo:(3,2)**	2.760	0.330	Fe(OH)2Co(OH)	0.84
**FeCo:(3,3)**	2.336	-0.093	Fe(OH)2	0.79
**FeCo:(3,4)**	2.381	-0.049	Fe(OH)2	0.83
**FeCo:(3,5)**	2.429	0.000	Fe(OH)2	0.78
**FeCo:(3,6)**	2.277	-0.153	Fe(OH)2	0.80
**FeCo:(4,0)**	4.523	2.094	Fe(OH)2	0.73
**FeCo:(4,2)**	3.110	0.680	Fe(OH)2	0.87
**FeCo:(4,4)**	2.518	0.088	Fe(OH)2	0.83
**FeCo:(4,6)**	2.590	0.160	Fe(OH)2Co(OH)	0.85
**FeCo:(5,1)**	2.446	0.017	Fe(OH)2	0.79
**FeCo:(5,2)**	2.271	-0.159	Fe(OH)2	0.82
**FeCo:(5,3)**	2.963	0.533	Fe(OH)2	0.77
**FeCo:(5,4)**	2.175	-0.254	Fe(OH)2	0.82
**FeCo:(5,5)**	2.579	0.149	Fe(OH)2	0.80
**FeCo:(6,1)**	2.911	0.481	Fe(OH)2	0.82
**FeCo:(6,3)**	2.508	0.079	Fe(OH)2Co(OH)	0.92
**FeCu:(0,2)**	3.744	0.436	Fe(OH)2	1.201
**FeCu:(0,4)**	3.816	0.507	Fe(OH)	0.49

**FeCu:(0,6)**	3.716	0.407	Fe(OH)	0.73
**FeCu:(1,1)**	4.358	1.050	Fe(OH)Cu(OH)2	0.92
**FeCu:(1,2)**	4.061	0.752	Fe(OH)2	1.08
**FeCu:(1,3)**	3.773	0.464	Fe(OH)	0.65
**FeCu:(1,4)**	3.222	-0.087	Fe(OH)	0.58
**FeCu:(1,5)**	3.033	-0.275	Fe(OH)	0.58
**FeCu:(1,6)**	3.137	-0.172	Fe(OH)	0.51
**FeCu:(2,1)**	3.520	0.211	Fe(OH)Cu(OH)2	0.97
**FeCu:(2,3)**	3.734	0.425	Fe(OH)	0.62
**FeCu:(2,5)**	3.522	0.213	Fe(OH)	0.49
**FeCu:(2,7)**	3.872	0.563	Fe(OH)	0.62
**FeCu:(3,0)**	4.059	0.750	Fe(OH)2	1.10
**FeCu:(3,1)**	3.652	0.343	Fe(OH)Cu(OH)	1.19
**FeCu:(3,2)**	3.718	0.409	Fe(OH)	0.58
**FeCu:(3,3)**	3.296	-0.013	Fe(OH)	0.43
**FeCu:(3,4)**	3.344	0.036	Fe(OH)	0.52
**FeCu:(3,5)**	3.348	0.040	Fe(OH)	0.54
**FeCu:(3,6)**	3.188	-0.120	Fe(OH)	0.63
**FeCu:(4,0)**	5.383	2.074	Fe(OH)	0.52
**FeCu:(4,2)**	3.995	0.687	Fe(OH)	0.64
**FeCu:(4,4)**	3.471	0.162	Bare	0.47
**FeCu:(4,6)**	3.453	0.144	Fe(OH)	0.76
**FeCu:(5,1)**	3.156	-0.153	Fe(OH)	0.59
**FeCu:(5,2)**	3.147	-0.161	Fe(OH)	0.68
**FeCu:(5,3)**	3.788	0.479	Fe(OH)	0.54
**FeCu:(5,4)**	3.028	-0.281	Fe(OH)	0.76
**FeCu:(5,5)**	3.390	0.082	Fe(OH)	0.60
**FeCu:(6,1)**	3.769	0.460	Fe(OH)	0.61
**FeCu:(6,3)**	3.331	0.022	Fe(OH)	0.68
**FeMn:(0,2)**	1.335	-0.813	Fe(OH)Mn(OH)2	0.77
**FeMn:(0,4)**	2.717	0.569	Fe(OH)Mn(OH)	0.47
**FeMn:(0,6)**	2.526	0.378	Fe(OH)Mn(OH)2	0.74
**FeMn:(1,1)**	4.072	1.925	Mn(OH)2	0.48
**FeMn:(1,2)**	2.845	0.697	Fe(OH)Mn(OH)	0.60
**FeMn:(1,3)**	2.583	0.435	Fe(OH)Mn(OH)2	0.66
**FeMn:(1,4)**	2.097	-0.050	Fe(OH)2Mn(OH)	0.62
**FeMn:(1,5)**	1.891	-0.257	Fe(OH)2Mn(OH)	0.60
**FeMn:(1,6)**	1.994	-0.154	Fe(OH)Mn(OH)2	0.63
**FeMn:(2,1)**	3.495	1.347	Fe(OH)2	0.24
**FeMn:(2,3)**	2.586	0.438	Fe(OH)Mn(OH)2	0.72
**FeMn:(2,5)**	2.386	0.239	Fe(OH)Mn(OH)2	0.58

**FeMn:(2,7)**	2.690	0.542	Fe(OH)Mn(OH)2	0.60
**FeMn:(3,0)**	2.675	0.527	Fe(OH)Mn(OH)2	0.60
**FeMn:(3,1)**	2.456	0.308	Fe(OH)Mn(OH)2	0.79
**FeMn:(3,2)**	2.573	0.425	Fe(OH)Mn(OH)2	0.65
**FeMn:(3,3)**	2.156	0.008	Fe(OH)Mn(OH)2	0.47
**FeMn:(3,4)**	2.147	0.000	Fe(OH)Mn(OH)2	0.64
**FeMn:(3,5)**	2.202	0.054	Fe(OH)Mn(OH)2	0.52
**FeMn:(3,6)**	2.015	-0.133	Fe(OH)Mn(OH)2	0.62
**FeMn:(4,0)**	4.169	2.022	Fe(OH)Mn(OH)	0.46
**FeMn:(4,2)**	2.802	0.654	Fe(OH)Mn(OH)2	0.64
**FeMn:(4,4)**	2.248	0.101	Fe(OH)Mn(OH)2	0.69
**FeMn:(4,6)**	2.290	0.142	Fe(OH)Mn(OH)2	0.69
**FeMn:(5,1)**	2.096	-0.052	Fe(OH)Mn(OH)2	0.49
**FeMn:(5,2)**	1.977	-0.170	Fe(OH)Mn(OH)2	0.69
**FeMn:(5,3)**	2.629	0.481	Fe(OH)Mn(OH)2	0.53
**FeMn:(5,4)**	1.883	-0.264	Fe(OH)Mn(OH)2	0.67
**FeMn:(5,5)**	2.256	0.108	Fe(OH)Mn(OH)2	0.63
**FeMn:(6,1)**	2.561	0.413	Fe(OH)Mn(OH)2	0.53
**FeMn:(6,3)**	2.169	0.021	Fe(OH)Mn(OH)2	0.71
**FeNi:(0,2)**	2.315	0.204	Fe(OH)	0.93
**FeNi:(0,4)**	2.612	0.502	Fe(OH)	0.51
**FeNi:(0,6)**	2.481	0.370	Fe(OH)	0.72
**FeNi:(1,1)**	3.710	1.599	Ni(OH)2	0.88
**FeNi:(1,2)**	2.739	0.629	Fe(OH)	0.55
**FeNi:(1,3)**	2.440	0.330	Fe(OH)	0.66
**FeNi:(1,4)**	1.961	-0.150	Fe(OH)	0.58
**FeNi:(1,5)**	1.783	-0.327	Fe(OH)	0.58
**FeNi:(1,6)**	1.891	-0.220	Fe(OH)	0.62
**FeNi:(2,1)**	3.113	1.002	Fe(OH)2Ni(OH)	1.17
**FeNi:(2,3)**	2.439	0.329	Fe(OH)	0.75
**FeNi:(2,5)**	2.315	0.204	Fe(OH)	0.60
**FeNi:(2,7)**	1.438	-0.672	Bare	1.07
**FeNi:(3,0)**	3.068	0.958	Fe(OH)	0.75
**FeNi:(3,1)**	2.604	0.493	Fe(OH)	0.76
**FeNi:(3,2)**	2.526	0.416	Fe(OH)	0.65
**FeNi:(3,3)**	2.084	-0.027	Fe(OH)	0.42
**FeNi:(3,4)**	2.141	0.031	Fe(OH)	0.64
**FeNi:(3,5)**	2.144	0.033	Fe(OH)	0.55
**FeNi:(3,6)**	1.994	-0.117	Bare	0.47
**FeNi:(4,0)**	4.445	2.335	Fe(OH)2	1.226
**FeNi:(4,2)**	2.846	0.736	Fe(OH)	0.62

**FeNi:(4,4)**	2.256	0.146	Fe(OH)	0.70
**FeNi:(4,6)**	2.282	0.171	Fe(OH)	0.65
**FeNi:(5,1)**	2.189	0.078	Fe(OH)	0.49
**FeNi:(5,2)**	2.049	-0.061	Fe(OH)	0.68
**FeNi:(5,3)**	2.702	0.591	Fe(OH)	0.53
**FeNi:(5,4)**	1.887	-0.223	Fe(OH)	0.65
**FeNi:(5,5)**	2.264	0.153	Fe(OH)	0.61
**FeNi:(6,1)**	2.656	0.545	Fe(OH)	0.63
**FeNi:(6,3)**	2.219	0.109	Fe(OH)	0.63
**FeZn:(0,2)**	3.567	0.926	Fe(OH)Zn(OH)	1.10
**FeZn:(0,4)**	3.188	0.548	Fe(OH)Zn(OH)	0.41
**FeZn:(0,6)**	3.103	0.462	Fe(OH)Zn(OH)	0.69
**FeZn:(1,1)**	4.880	2.239	Fe(OH)2Zn(OH)	0.78
**FeZn:(1,2)**	3.615	0.975	Fe(OH)Zn(OH)	0.65
**FeZn:(1,3)**	3.257	0.616	Fe(OH)Zn(OH)	0.84
**FeZn:(1,4)**	2.604	-0.036	Fe(OH)Zn(OH)	0.56
**FeZn:(1,5)**	2.400	-0.241	Fe(OH)Zn(OH)	0.56
**FeZn:(1,6)**	2.501	-0.139	Fe(OH)Zn(OH)	0.63
**FeZn:(2,1)**	3.580	0.939	Fe(OH)2Zn(OH)	0.97
**FeZn:(2,3)**	3.176	0.535	Fe(OH)Zn(OH)	1.00
**FeZn:(2,5)**	2.884	0.244	Fe(OH)Zn(OH)	0.58
**FeZn:(2,7)**	3.237	0.597	Fe(OH)Zn(OH)	0.55
**FeZn:(3,0)**	3.368	0.727	Fe(OH)Zn(OH)	0.96
**FeZn:(3,1)**	3.039	0.398	Fe(OH)Zn(OH)	1.05
**FeZn:(3,2)**	3.046	0.405	Fe(OH)Zn(OH)	0.82
**FeZn:(3,3)**	2.661	0.020	Fe(OH)Zn(OH)	0.55
**FeZn:(3,4)**	2.672	0.031	Fe(OH)Zn(OH)	0.62
**FeZn:(3,5)**	2.690	0.049	Fe(OH)Zn(OH)	0.53
**FeZn:(3,6)**	2.507	-0.133	Fe(OH)Zn(OH)	0.61
**FeZn:(4,0)**	4.554	1.914	Fe(OH)Zn(OH)	0.40
**FeZn:(4,2)**	3.315	0.674	Fe(OH)Zn(OH)	0.70
**FeZn:(4,4)**	2.802	0.161	Fe(OH)Zn(OH)	0.62
**FeZn:(4,6)**	2.778	0.137	Fe(OH)Zn(OH)	0.71
**FeZn:(5,1)**	2.332	-0.308	Fe(OH)2	0.63
**FeZn:(5,2)**	2.390	-0.251	Fe(OH)Zn(OH)	0.64
**FeZn:(5,3)**	3.063	0.422	Fe(OH)Zn(OH)	0.58
**FeZn:(5,4)**	2.299	-0.342	Fe(OH)Zn(OH)	0.66
**FeZn:(5,5)**	2.682	0.042	Fe(OH)Zn(OH)	0.67
**FeZn:(6,1)**	3.040	0.399	Fe(OH)Zn(OH)	0.55
**FeZn:(6,3)**	2.599	-0.042	Fe(OH)Zn(OH)	0.75
**FePt:(0,2)**	3.263	0.390	Fe(OH)2	1.18

**FePt:(0,4)**	3.613	0.740	Fe(OH)2	1.218
**FePt:(0,6)**	3.409	0.536	Fe(OH)	0.74
**FePt:(1,1)**	4.874	2.001	Fe(OH)Pt(OH)	1.14
**FePt:(1,2)**	3.802	0.929	Fe(OH)2	1.06
**FePt:(1,3)**	3.488	0.615	Fe(OH)2	1.214
**FePt:(1,4)**	2.897	0.024	Fe(OH)	0.60
**FePt:(1,5)**	2.630	-0.243	Fe(OH)	0.59
**FePt:(1,6)**	2.753	-0.119	Fe(OH)	0.63
**FePt:(2,1)**	3.887	1.015	Fe(OH)2	0.89
**FePt:(2,3)**	3.451	0.578	Fe(OH)2	1.20
**FePt:(2,5)**	3.209	0.336	Bare	0.47
**FePt:(2,7)**	3.545	0.672	Fe(OH)2	1.216
**FePt:(3,0)**	3.423	0.550	Fe(OH)2	1.13
**FePt:(3,1)**	3.229	0.356	Fe(OH)2	1.10
**FePt:(3,2)**	3.245	0.373	Bare	0.49
**FePt:(3,3)**	2.968	0.095	Fe(OH)2	1.221
**FePt:(3,4)**	2.915	0.042	Fe(OH)	0.64
**FePt:(3,5)**	2.952	0.079	Fe(OH)2	1.22
**FePt:(3,6)**	2.732	-0.141	Fe(OH)	0.64
**FePt:(4,0)**	4.862	1.989	Fe(OH)2	1.228
**FePt:(4,2)**	3.531	0.658	Fe(OH)	0.64
**FePt:(4,4)**	3.018	0.145	Fe(OH)	0.71
**FePt:(4,6)**	3.056	0.183	Fe(OH)	0.66
**FePt:(5,1)**	2.729	-0.144	Fe(OH)2	1.218
**FePt:(5,2)**	2.577	-0.296	Fe(OH)	0.69
**FePt:(5,3)**	3.419	0.546	Fe(OH)2	1.220
**FePt:(5,4)**	2.485	-0.388	Bare	0.48
**FePt:(5,5)**	2.976	0.104	Fe(OH)2	1.220
**FePt:(6,1)**	3.323	0.450	Fe(OH)2	1.226
**FePt:(6,3)**	2.871	-0.002	Bare	0.50

Table S2. Effect of spin state on ORR activity, using FeCu-N-C (0,6) DAC as a representative example.

**Spin state**	**ΔG_1'**	**ΔG_1**	**ΔG_2**	**ΔG_3**	**ΔG_4**
**High**	-0.25	-0.96	-1.62	-1.37	-0.72
**Medium**	-0.30	-0.86	-1.53	-1.51	-0.73
**Low**	-0.21	-0.97	-1.63	-1.43	-0.68

Table S3. Comparison of Gibbs free energies of formation (eV) for the 9 FeMn-N-C (0,6) DAC phases calculated using GGA-PBE and HSE06 functionals.

Phase	GGA-PBE	HSE06
**Bare**	0.000	0.000
**Fe(OH)**	0.304	0.297
**Mn(OH)**	0.224	0.289
**Fe(OH)Mn(OH)**	0.527	0.570
**Fe(OH)2**	1.008	1.084
**Mn(OH)2**	0.699	0.740
**Fe(OH)2Mn(OH)**	1.156	1.164
**Fe(OH)Mn(OH)2**	1.041	0.989
**Fe(OH)2Mn(OH)2**	1.728	1.859

Table S4. Summary of the number of each type of active site identified across all 186 DACs.

Structure	Mn	Zn	Co	Ni	Cu	Pt
**Bare**	0	0	0	2	1	4
**Fe(OH)**	0	0	0	27	26	10
**M(OH)**	0	0	0	0	0	0
**Fe(OH)M(OH)**	3	28	0	0	0	1
**Fe(OH)2**	1	1	19	2	4	16
**M(OH)2**	1	0	0	0	0	0
**Fe(OH)2M(OH)**	2	2	9	0	0	0
**Fe(OH)M(OH)2**	24	0	3	0	0	0
**Fe(OH)2M(OH)2**	0	0	0	0	0	0

Table S5. A summary of formation energies, relative stabilities, active site under reaction conditions, and limiting potentials for the 29 DACs that outperform Pt.

DAC	Formation Energy (eV)	Relative Stability (eV)	Active Site	Limiting Potential (V)
**FeCo:(0,6)**	2.821	0.392	Fe(OH)2Co(OH)	0.82
**FeCo:(1,1)**	3.859	1.429	Fe(OH)2	0.86
**FeCo:(1,3)**	2.719	0.290	Fe(OH)2	0.83
**FeCo:(1,4)**	2.251	-0.178	Fe(OH)2Co(OH)	0.80
**FeCo:(1,5)**	2.109	-0.321	Fe(OH)2Co(OH)	0.81
**FeCo:(1,6)**	2.207	-0.222	Fe(OH)2Co(OH)	0.83
**FeCo:(2,3)**	2.724	0.294	Fe(OH)2Co(OH)	0.88
**FeCo:(2,5)**	2.616	0.187	Fe(OH)2	0.83
**FeCo:(3,1)**	2.957	0.528	Fe(OH)Co(OH)2	0.80
**FeCo:(3,2)**	2.760	0.330	Fe(OH)2Co(OH)	0.84
**FeCo:(3,4)**	2.381	-0.049	Fe(OH)2	0.83
**FeCo:(3,6)**	2.277	-0.153	Fe(OH)2	0.80
**FeCo:(4,2)**	3.110	0.680	Fe(OH)2	0.87
**FeCo:(4,4)**	2.518	0.088	Fe(OH)2	0.83
**FeCo:(4,6)**	2.590	0.160	Fe(OH)2Co(OH)	0.85
**FeCo:(5,2)**	2.271	-0.159	Fe(OH)2	0.82
**FeCo:(5,4)**	2.175	-0.254	Fe(OH)2	0.82
**FeCo:(6,1)**	2.911	0.481	Fe(OH)2	0.82
**FeCo:(6,3)**	2.508	0.079	Fe(OH)2Co(OH)	0.92
**FeCu:(1,1)**	4.358	1.050	Fe(OH)Cu(OH)2	0.92
**FeCu:(2,1)**	3.520	0.211	Fe(OH)Cu(OH)2	0.97
**FeNi:(0,2)**	2.315	0.204	Fe(OH)	0.93
**FeNi:(1,1)**	3.710	1.599	Ni(OH)2	0.88
**FeZn:(1,3)**	3.257	0.616	Fe(OH)Zn(OH)	0.84
**FeZn:(2,1)**	3.580	0.939	Fe(OH)2Zn(OH)	0.97
**FeZn:(3,0)**	3.368	0.727	Fe(OH)Zn(OH)	0.96
**FeZn:(3,2)**	3.046	0.405	Fe(OH)Zn(OH)	0.82
**FePt:(2,1)**	3.887	1.015	Fe(OH)2	0.89
**FeMn:(3,1)**	2.456	0.308	Fe(OH)Mn(OH)2	0.79

REFERENCES

(1) Kresse, G.; Furthmüller, J. Efficiency of Ab-Initio Total Energy Calculations for Metals and Semiconductors Using a Plane-Wave Basis Set. *Computational Materials Science* **1996**, *6* (1), 15–50. https://doi.org/10.1016/0927-0256(96)00008-0.

(2) Kresse, G.; Furthmüller, J. Efficient Iterative Schemes for *Ab Initio* Total-Energy Calculations Using a Plane-Wave Basis Set. *Phys. Rev. B* **1996**, *54* (16), 11169–11186. https://doi.org/10.1103/PhysRevB.54.11169.

(3) Perdew, J. P.; Burke, K.; Ernzerhof, M. Generalized Gradient Approximation Made Simple. *Phys. Rev. Lett.* **1996**, *77* (18), 3865–3868. https://doi.org/10.1103/PhysRevLett.77.3865.

(4) Blöchl, P. E. Projector Augmented-Wave Method. *Phys. Rev. B* **1994**, *50* (24), 17953–17979. https://doi.org/10.1103/PhysRevB.50.17953.

(5) Kattel, S.; Wang, G. A Density Functional Theory Study of Oxygen Reduction Reaction on Me–N4 (Me = Fe, Co, or Ni) Clusters between Graphitic Pores. *J. Mater. Chem. A* **2013**, *1* (36), 10790. https://doi.org/10.1039/c3ta12142a.

(6) Kattel, Shyam; Plamen Atanassov; Boris Kiefer. Stability, Electronic and Magnetic Properties of in-Plane Defects in Graphene: A First-Principles Study. *The Journal of Physical Chemistry C* **2012**, *116* (14), 8161–8166.

(7) Shang, Yan Zhao; Jing-xiang Zhao; Hong Wu; Qing-hai Cai; Xiao-guang Wang; Xuan-zhang Wang. Chemical Functionalization of Pyridine-like and Prophyrin-like Nitrogen-Doped Carbon (CN x) Nanotubes with Transition Metal (TM) Atoms: A Theoretical Study. *Theoretical Chemistry Accounts 127* (5), 727–733.

(8) Hansen, H. A.; Rossmeisl, J.; Nørskov, J. K. Surface Pourbaix Diagrams and Oxygen Reduction Activity of Pt, Ag and Ni(111) Surfaces Studied by DFT. *Phys. Chem. Chem. Phys.* **2008**, *10* (25), 3722. https://doi.org/10.1039/b803956a.

(9) Nørskov, J. K.; Rossmeisl, J.; Logadottir, A.; Lindqvist, L.; Kitchin, J. R.; Bligaard, T.; Jónsson, H. Origin of the Overpotential for Oxygen Reduction at a Fuel-Cell Cathode. *J. Phys. Chem. B* **2004**, *108* (46), 17886–17892. https://doi.org/10.1021/jp047349j.

(10) Sun, F.; Li, F.; Tang, Q. Spin State as a Participator for Demetalation Durability and Activity of Fe–N–C Electrocatalysts. *J. Phys. Chem. C* **2022**, *126* (31), 13168–13181. https://doi.org/10.1021/acs.jpcc.2c03518.

(11) Anderson, A. B. Insights into Electrocatalysis. *Physical Chemistry Chemical Physics* **2012**, *14* (4), 1330–1338. https://doi.org/10.1039/C2CP23616H.

(12) Anderson, Alfred B.; Jamal Uddin; Ryoske Jinnouchi. Solvation and Zero-Point Energy Effects on OH (Ads) Reduction on Pt(111) Electrodes. *The Journal of Physical Chemistry C* **2010**, *114* (35), 14946–14952. https://doi.org/10.1021/jp1041734.

(13) Holby, E. F.; Taylor, C. D. Activity of N-Coordinated Multi-Metal-Atom Active Site Structures for Pt-Free Oxygen Reduction Reaction Catalysis: Role of *OH Ligands. *Sci Rep* **2015**, *5* (1), 9286. https://doi.org/10.1038/srep09286.

(14) Kulkarni, A.; Siahrostami, S.; Patel, A.; Nørskov, J. K. Understanding Catalytic Activity Trends in the Oxygen Reduction Reaction. *Chem. Rev.* **2018**, *118* (5), 2302–2312. https://doi.org/10.1021/acs.chemrev.7b00488.
